# Digital twins elucidate critical role of T_scm_ in clinical persistence of TCR-engineered cell therapy

**DOI:** 10.1038/s41540-024-00335-7

**Published:** 2024-01-26

**Authors:** Louis R. Joslyn, Weize Huang, Dale Miles, Iraj Hosseini, Saroja Ramanujan

**Affiliations:** Genentech Inc., South San Francisco, CA USA

**Keywords:** Immunology, Computer modelling, Cancer, Biomarkers

## Abstract

Despite recent progress in adoptive T cell therapy for cancer, understanding and predicting the kinetics of infused T cells remains a challenge. Multiple factors can impact the distribution, expansion, and decay or persistence of infused T cells in patients. We have developed a novel quantitative systems pharmacology (QSP) model of TCR-transgenic T cell therapy in patients with solid tumors to describe the kinetics of endogenous T cells and multiple memory subsets of engineered T cells after infusion. These T cells undergo lymphodepletion, proliferation, trafficking, differentiation, and apoptosis in blood, lymph nodes, tumor site, and other peripheral tissues. Using the model, we generated patient-matched digital twins that recapitulate the circulating T cell kinetics reported from a clinical trial of TCR-engineered T cells targeting E7 in patients with metastatic HPV-associated epithelial cancers. Analyses of key parameters influencing cell kinetics and differences among digital twins identify stem cell-like memory T cells (T_scm_) cells as an important determinant of both expansion and persistence and suggest that T_scm_-related differences contribute significantly to the observed variability in cellular kinetics among patients. We simulated in silico clinical trials using digital twins and predict that T_scm_ enrichment in the infused product improves persistence of the engineered T cells and could enable administration of a lower dose. Finally, we verified the broader relevance of the QSP model, the digital twins, and findings on the importance of T_scm_ enrichment by predicting kinetics for two patients with pancreatic cancer treated with KRAS G12D targeting T cell therapy. This work offers insight into the key role of T_scm_ biology on T cell kinetics and provides a quantitative framework to evaluate cellular kinetics for future efforts in the development and clinical application of TCR-engineered T cell therapies.

## Introduction

The past decade has witnessed rapid progress in the field of adoptive T cell therapy, including the approval of multiple chimeric antigen receptor (CAR) T cell therapies in hematological cancers^1^. CAR-T cells, engineered to target surface antigens on tumor cells to drive tumor eradication, is a therapy that can result in long-term remission alongside T cell persistence in the most successful cases. Another approach is TCR-engineered T cells, designed to recognize MHC-presented antigen to enable targeting of intracellular proteins^2^. Recently, both CAR- and TCR-engineered T cells have shown at least partial efficacy against solid tumors^2,3^, expanding on initial successes in hematological malignancies.

As a ‘living drug’, T cell therapy products are complex, heterogeneous, and can proliferate after infusion^4^. Therefore, understanding the cellular kinetics and pharmacology of T cell therapies can be difficult compared to protein-based therapeutics, for which molecule properties are more uniform and pharmacokinetics are better characterized. Cellular kinetics of T cell therapies tend to follow four phases: 1) distribution – a steep decline in circulating T cell concentrations as cells traffic to tissue immediately following infusion, 2) expansion – a rapid increase in T cell concentrations following distribution and 3) contraction – a decline in cell concentrations following expansion and 4) decline or persistence – a slow decay in cell concentrations that can last months to years^4,5^. Each phase results from a combination of biological processes such as T cell proliferation, trafficking, differentiation, activation, apoptosis, exhaustion and anergy^6,7^, however their relative contributions to different aspects of cellular kinetics have yet to be deconvolved. Furthermore, the infused product can include T cells with varying levels of memory differentiation, activation, plasticity, or even exhaustion^8–10^, and the impact of these variables is also not well characterized.

Understanding the cellular kinetics of TCR-engineered T cell therapies in solid tumor indications is crucial to ensure T cell expansion, migration, antigen recognition, tumor regression, and maintenance of long-term surveillance to improve the efficacy of these therapies and prevent tumor recurrence. As a complementary approach to in vitro and in vivo experiments, mathematical and in silico computational models have been developed to study the cellular kinetics of T cell therapies. These models typically use nonlinear differential equations to describe and simulate the dynamics of T cell therapies and have focused on CAR T cell therapies in hematological malignancies^11–13^. However, no models have been developed to describe TCR-engineered T cell kinetics for solid tumors and only a few have attempted to capture the various T cell memory phenotypes present in T cell therapies^7,11,14^.

In this work, we present a quantitative systems pharmacology (QSP) model that represents fundamental mechanisms of T cell turnover, differentiation, and trafficking to describe the multiphasic kinetics of TCR-engineered T cells, including different T cell memory phenotypes and endogenous T cells in multiple physiological compartments following lymphodepletion and T cell infusion. We use the model to reproduce and analyze the cellular kinetics data reported in a clinical study of TCR-engineered T cells targeting neoantigen E7 in patients with HPV-associated epithelial cancer^15^. Given the small size of the study and the high degree of interpatient variability, we apply a recently developed digital twin methodology^16^ to create “individualized” virtual patients that reproduce observed cellular kinetics from each study patient. We perform quantitative analysis of the digital twins to identify mechanisms that impact cellular kinetics and explore how dose amount and composition influence cell expansion and persistence. We also show that the resulting model, digital twins, and findings are more broadly applicable by predicting kinetics from a clinical trial on KRAS G12D targeting T cell therapy^17^. The model and analysis presented provide insight into biological processes governing the kinetics of TCR-engineered T cell therapies, highlight an important role of T_scm_ biology and provide a quantitative framework to inform future efforts in the development and clinical application of TCR-engineered T cell therapies.

## Results

### Digital twins capture kinetics of multiple T cell phenotypes at different dose levels and compositions

**Fig. 1:**
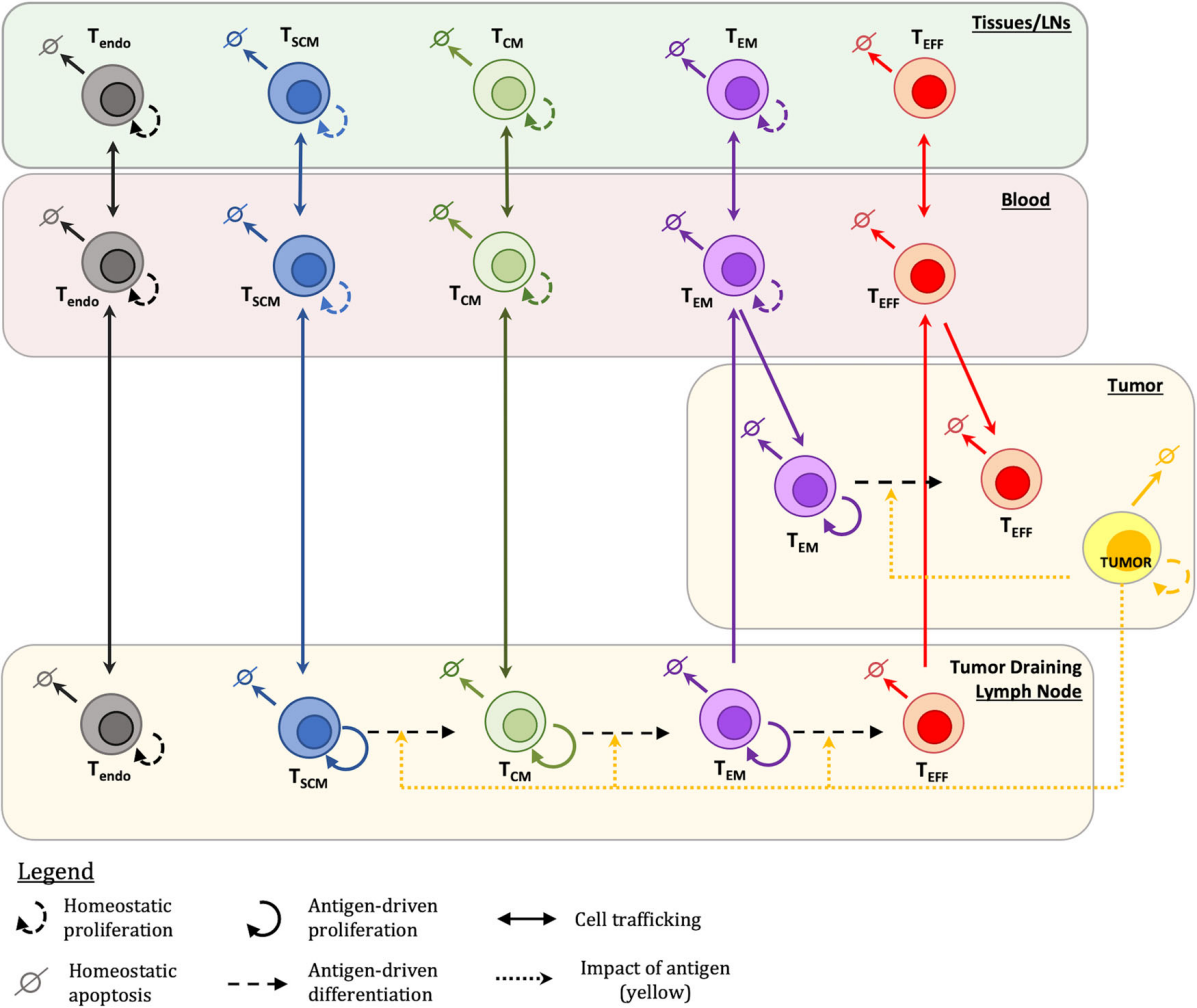
QSP model describes dynamics of T cell phenotypes across multiple physiological compartments. The QSP model describes the in vivo dynamics of multiple T cell phenotypes following treatment with TCR-engineered T cells. The model includes four T cell phenotypes, T_scm_, T_cm_, T_em_, and T_eff_, across blood, tumor, tumor-draining lymph node, and other tissues/lymph nodes compartments. Endogenous T cells (T_endo_) are present in all non-tumor compartments. Baseline proliferation (except T_eff_), apoptosis, and trafficking between compartments are represented for all T cells. Antigen-driven proliferation and engineered T cell differentiation is modeled in the tumor and tumor-draining lymph node, and T_em_ and T_eff_ traffic through the blood to the tumor compartment.

**Fig. 2:**
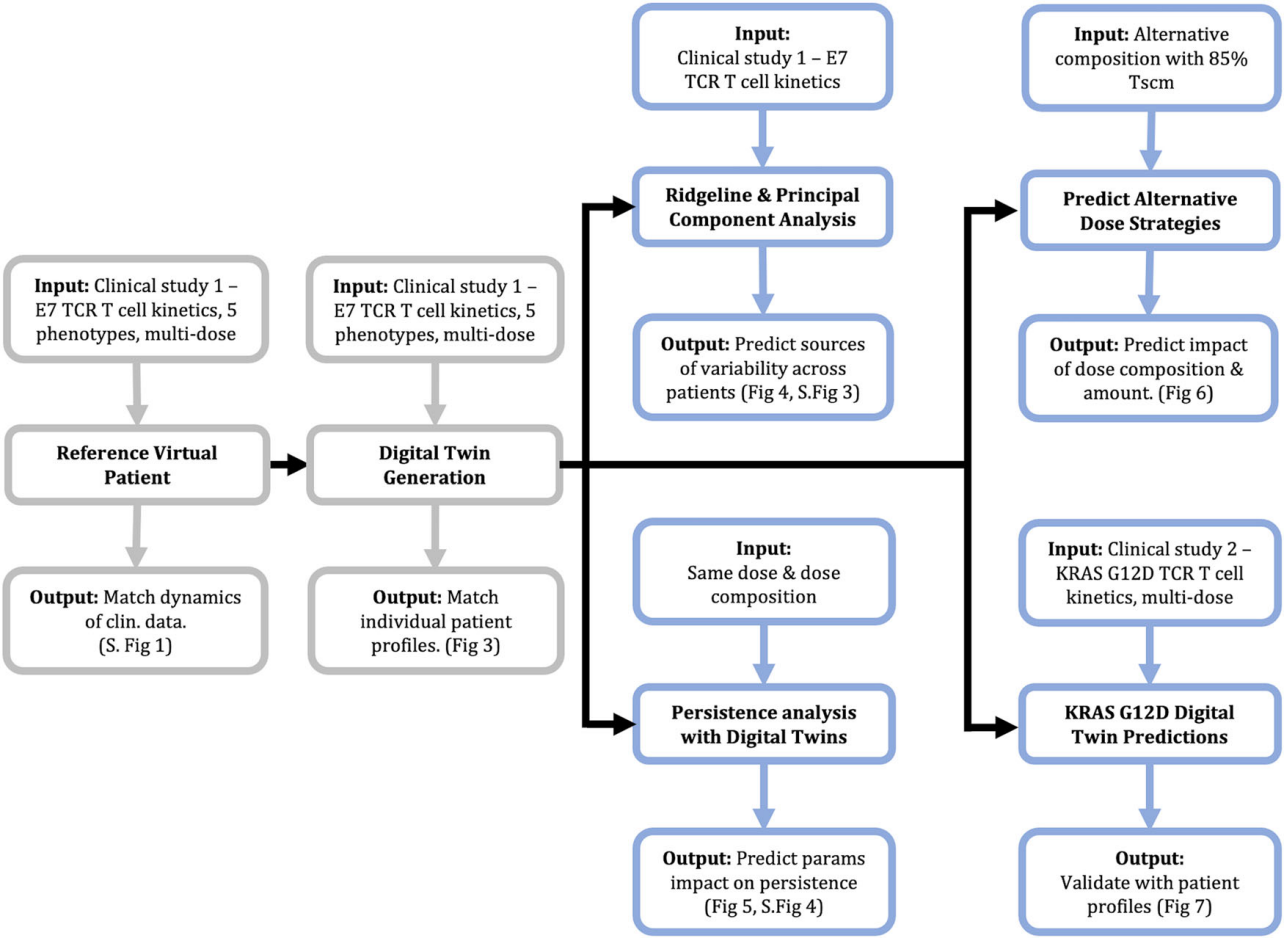
Workflow of T cell therapy QSP model development, calibration, and analysis. The QSP model represents cellular kinetics of T cells in blood, tissues, tumor-draining lymph node and tumor for 5 T cell phenotypes. Model calibration used clinical data of E7 targeting TCR-engineered T cells to generate a ‘reference virtual patient’, to demonstrate that generating a fit was feasible but cannot capture all observed variability in cellular kinetics across patients. Subsequently, we used the same dataset to generate a set of digital twins, wherein 10 digital twins were selected to match each patient in the clinical trial. We use the digital twins to make predictions for alternate dosing strategies and performed parameter analyses to gain insight into biological mechanisms that might differ from patient to patient or drive persistence across time. We simulated the digital twins with an alternative dose strategy to predict the impact of dose composition and dose amount on cellular kinetics. Finally, we compare the digital twin predictions for a separate TCR-engineered T cell therapy targeting KRAS G12D in pancreatic cancer patients.

The QSP model represents fundamental mechanisms of T cell biology to describe the cellular kinetics of endogenous CD3 + T cells (T_endo_) and CD8 + TCR-engineered T cells (stem cell-like memory T cells -T_scm_, central memory T cells - T_cm_, effector memory T cells - T_em_, effector T cells - T_eff_) in multiple physiological compartments following lymphodepletion and T cell infusion (Fig. 1, see Methods & Fig. 2 for details). We first calibrated the model using gQSPSim workflows^18^ to generate a representative “reference virtual patient” across all dose cohorts that recapitulates the overall quantitative data on cellular kinetics after therapy with autologous T cells engineered to target the neoantigen E7 in metastatic HPV-associated epithelial cancer patients (Supplementary Fig. 1B)^15^. We selected to calibrate the model to this publicly available clinical data because the study provided dose amounts, dose compositions and cellular kinetics for each patient (see Fig. 2A in Nagarsheth et al. which displays cellular kinetics across time for each patient^15^). Simulation of the reference patient at each dose level (low dose - 10^9^ edited cells, middle dose - 10^10^ edited cells, high dose - 10^11^ edited cells) reproduces the observed clinical cellular kinetics in blood. All subsets of TCR-engineered T cells characterized in the study (T_scm_, T_cm_, T_em_, and T_eff_ cells) exhibit distribution, expansion and decay over time, except in the low dose group, in which neither the observed data nor the reference virtual patient shows expansion of the T cells. These results verify that the model and reference virtual patient can quantitatively describe the dynamics of all TCR-engineered T cell subsets as well as the endogenous T cells, as a function of total infused T cell dose (Supplementary Fig. 1), but may not be able to capture all the variability present in the data.

As the cellular kinetics show a high degree of interpatient variability within and across dose cohorts (Supplementary Fig. 1A), we employed a digital twin approach (Supplementary Fig. 2). The digital twin methodology is a relatively new approach for calibrating QSP models when there is limited observed data for different clinical dosing scenarios and a high degree of variability^16^. For each patient, we screened thousands of virtual patients to select 10 digital twins, each of which reflects a unique combination of underlying biological parameter values to closely match the observed patient kinetics when simulated with the corresponding clinically administered dose amount and composition (see Supplementary Methods for digital twin generation and workflow). Given the uncertainty in biological parameters, the use of multiple digital twins per patient allows us to explicitly account for alternative parameterizations of the underlying biology that are consistent with the observed data, but may yield divergent predictions under untested protocols and different dosing regimens or cell composition of the infused product.

**Fig. 3:**
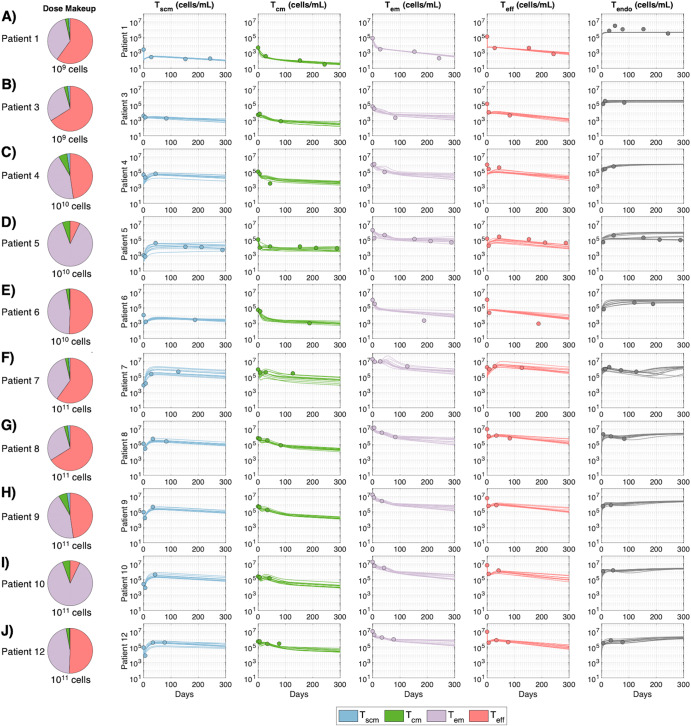
Digital twins capture the variable cellular kinetics of patients treated with TCR-engineered T cells. The digital twins replicate the multiphasic cellular kinetics in blood following treatment with HPV-16 E7 targeting TCR-engineered T cells. Each column displays experimental measurements in blood: either T_scm_, T_cm_, T_em_, or T_eff_ TCR-engineered T cells, endogenous T cells (T_endo_). Every row displays cell measurements in units of cells/mL for an individual patient. **A**, **B** Patients 1 and 3 received 10^9^ cells, (**C**–**E**) patients 4, 5, and 6 received 10^10^ cells and (**F**–**J**) all other patients received 10^11^ cells. Data points represent the experimental data across time and curves are the 10 digital twins that best matched the experimental data for that patient. Patients 2 and 11 were removed due to a lack of reported data.

The digital twins successfully capture the distinct cellular kinetics of each patient in response to distinct dose amounts and compositions of TCR-engineered T cells (Fig. 3, patient IDs 2 & 11 were removed from the original dataset due to a lack of data). Like the reference patient, the digital twins capture the general differences between cohorts, with minimal cell expansion in the patients of the low dose group (IDs 1, 3) and greater expansion of T_scm_ and T_eff_ cells in the patients of the high dose cohort (IDs 7, 8, 9, 10, 12). The digital twins additionally capture within-group variability; for example, in the middle dose group, simulations reproduce the higher cell numbers and different kinetic profiles in patient 5 compared to those of patients 4 and 6.

Taken together, the superset of all digital twins forms a virtual population that represents the 10 patients in the clinical study. The virtual population can be used to project cellular kinetics for the 10 clinical patients under different TCR-engineered T cell therapy regimens and compositions and to dissect the biological processes driving the observed and predicted kinetics and interpatient variability.

### Proliferation and trafficking of early memory T cell phenotypes explain differing cellular kinetics across and within dose cohorts

**Fig. 4:**
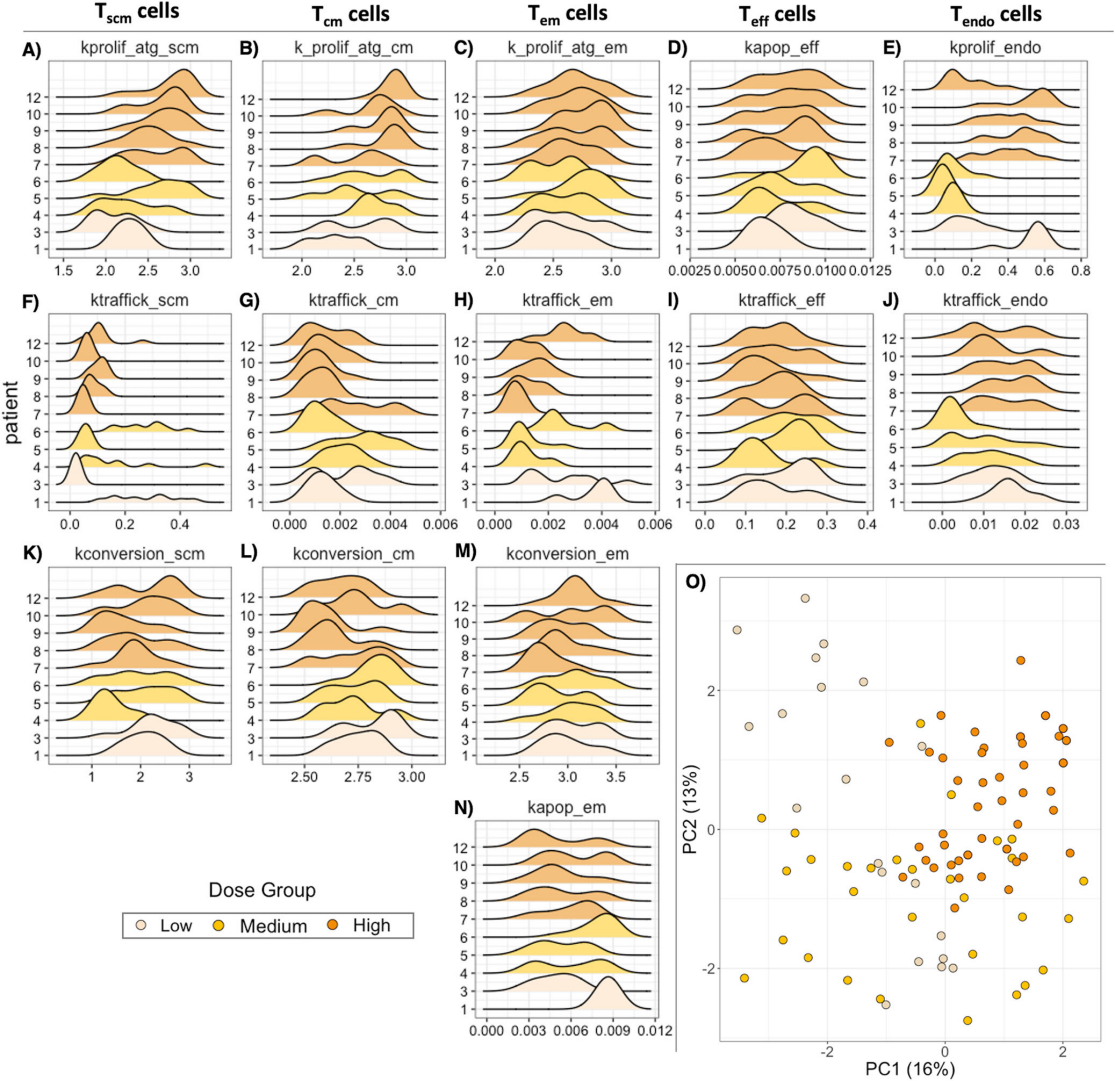
Parameter space ridgeline plots and principal component analysis of digital twins identify sources of variability between patients and across dose groups. **A**–**N** Ridgeline plots display the distributions for 14 parameters (1 subplot per parameter) across the digital twins of each clinical patient. For each subplot, individual patient IDs are listed along the y axis, and the range of potential parameter values are listed along the x axis. Each individual density plot represents the parameter values of the 10 digital twins that best matched the observed clinical data for that patient and is colored according to the dose group (tan – low dose, yellow – middle dose, orange - high dose). **O** The PCA plot in the lower right panel illustrates variability across principal components of parameter space (PC1 vs PC2). Each dot is an individual digital twin, colored according to the dose group, with a total of 100 digital twins represented.

The digital twins are defined by distinct inferred parameterizations of the underlying biological processes governing cellular kinetics. Ridgeline plots (Fig. 4A–N) and principal component analysis (PCA) (Fig. 4O, Supplementary Fig. 3) were separately used to visualize the parameter space for digital twins of each patient and to compare these across patients. Parameters that vary between clinical patients highlight potential underlying biology that is important for capturing their differential kinetic profiles.

The ridgeline plots (Fig. 4A–N) show consistent distributions across patients for many parameters, such as antigen-driven T_em_ proliferation (Fig. 4C) and T_em_ conversion (Fig. 4M); these processes do not appear to drive interpatient differences in cellular kinetics. However, the rate constant for antigen-driven proliferation of T_scm_ cells (i.e., kprolif_atg_scm subplot - Fig. 4A) varies both within and across dose groups. Within the mid-dose group, the digital twins for patient 5 skew to higher T_scm_ proliferation rate constants, similar to values in the high dose cohort, whereas the digital twins for patients 4 and 6 have lower T_scm_ proliferation rate constants, comparable to values in the low dose cohort. Correspondingly, patient 5 exhibits higher cell counts than patients 4 and 6, suggesting that the propensity of antigen-driven T_scm_ proliferation is a driver of interpatient variability in T cell numbers. Other parameters and processes with high inter-patient variability include the trafficking rate constants of T_scm_, T_cm_, and T_em_ (Fig. 4F–H) and the proliferation rate constants of T_cm_ (Fig. 4B) and endogenous cells (Fig. 4E). Thus, these biological processes appear to be important determinants of cell kinetics and heterogeneity.

As mentioned above, some parameters show clear inter-cohort variability. This is further evident by plotting the parameter values within the context of a PCA plot (Fig. 4O), where we observe minimal overlap along the first principal component (PC1) between the low and high dose groups (tan vs. orange dots, Fig. 4). In the PCA, parameters that govern proliferation and trafficking of T cells are important contributors of variability across dose cohorts (see Supplementary Fig. 3 for contribution of each parameter to PC1 and PC2). In agreement, the ridgeline plots also reveal higher T_scm_ and T_cm_ proliferation at high vs. low dose. These trends suggest an interplay between dose and early memory T cell proliferation, beyond the effects explicitly included in the model.

### Stratification of digital twins in a virtual clinical trial provides insight into key determinants of T cell persistence

**Fig. 5:**
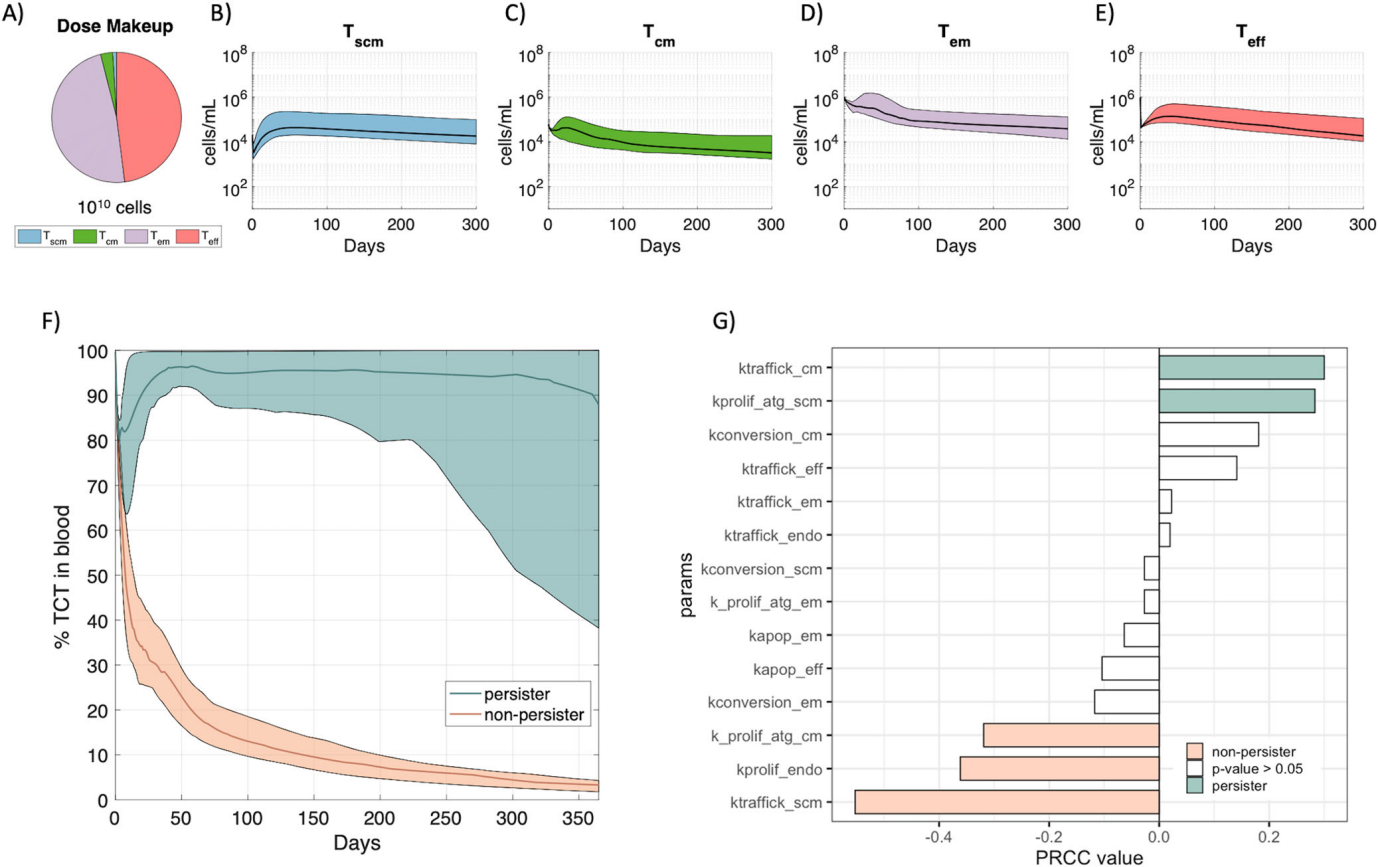
Biological variability (patient-specific) impacts cellular kinetics of TCR-engineered T cell therapies leading to persister or non-persister outcomes. **A** The entire set of digital twins were simulated with a dose of 10^10^ TCR-engineered T cells and a dose composition of 1% T_scm_, 3% T_cm_, 48% T_em_ and 48% T_eff_ cells. **B**–**E** The cellular kinetics of each T cell phenotype across time. The bands represent the 25th and 75th percentiles of all digital twins and the black line displays the median. **F** TCR-engineered T cells, as a percentage of total T cells in the blood, were delineated by persister (teal) or non-persister (peach) outcomes. The bands represent the 25th and 75th percentiles of each subgroup and the colored line displays the median. **G** Bar plot of Partial Rank Correlation Coefficient (PRCC) scores for the relationship between each parameter (along y axis) and outcome (persister vs non-persister). White bars represent parameters that were statistically insignificant.

While the above analysis illustrates the biological variability inherent across the digital twin population, the digital twins also allow the prediction of the impact of interpatient biological variability on T cell persistence over time. A virtual clinical trial was simulated where all the digital twins received a mid-dose of 10^10^ TCR-engineered T cells, at a composition of 1% T_scm_, 3% T_cm_, 48% T_em_ and 48% T_eff_ cells (Fig. 5A). This dose was selected as it is a representative dose composition from the E7 clinical trial. Figure 5B–E displays the simulated kinetics (median and interquartile range) of the different T cell phenotypes across the virtual population. At this dose amount and composition, the T_scm_ and T_eff_ cells (Fig. 5B, C) undergo distribution and subsequent expansion prior to decline across all the digital twins, while less expansion is predicted for T_cm_ and T_em_ cells (Fig. 5C, D). The predicted responses range over nearly an order of magnitude for each T cell phenotype, highlighting anticipated interpatient variability at a fixed dose composition.

Given the reported relationship between cell expansion in the first couple of months and treatment efficacy in hematological malignancies^5,14^, we stratified the digital twins based on frequency of TCR-engineered T cells (formulated as total TCR-engineered T cells divided by total T cells) in blood at day 50; those with a majority of TCR-engineered T cells in the blood exhibited persistence over time and hence were classified as ‘persisters’ under this treatment regimen, and the rest showed decline over time and were classified as ‘non-persisters’ (Fig. 5F).

We then performed a global sensitivity analysis to propose biological processes that might be driving persister vs. non-persister outcomes. Rate constants for T_scm_ trafficking and proliferation, T_endo_ proliferation, and T_cm_ proliferation and trafficking were identified as key drivers of persister or non-persister outcomes (Partial Rank Correlation Coefficient values plotted in Fig. 5G). Interestingly, these parameters were also identified in the inter-individual variability analysis above. We note that non-linear relationships can exist between parameters identified in such an analysis. For example, since overall T_scm_ expansion relies on both traffic to and proliferation within the tumor draining lymph node, digital twins with lower T_scm_ trafficking rate constants require greater T_scm_ proliferation rate constants to exhibit persistence and vice versa; thus, differences in these parameters between persisters and non-persisters are more evident in a bivariate plot (Supplementary Fig. 4). Altogether, these parameters appear to drive interpatient variability in cell persistence.

### Altering dose composition changes cellular kinetics of engineered-TCR T cell therapies

**Fig. 6:**
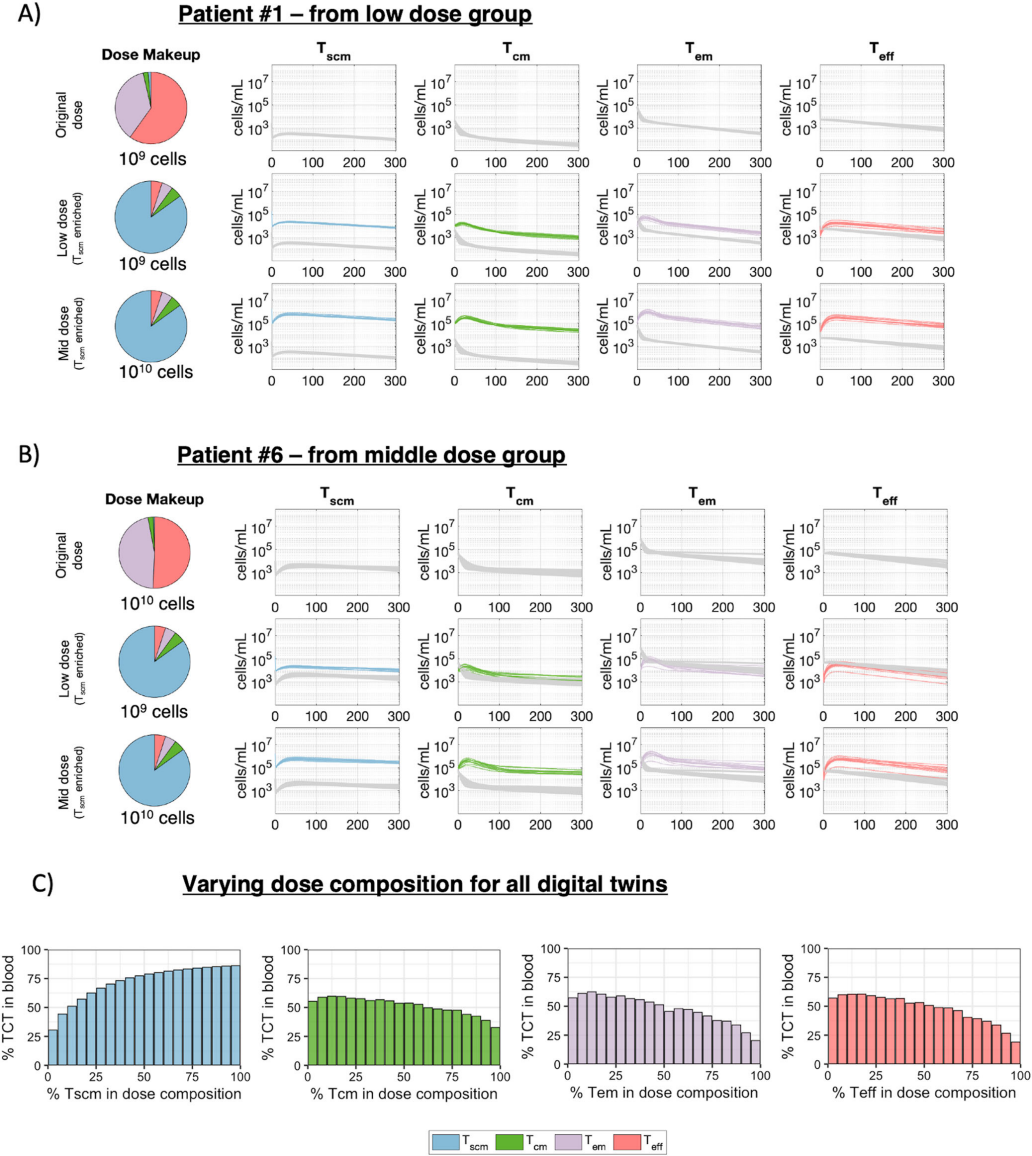
Dose composition impacts cellular kinetics of TCR-engineered T cells. Two sets of digital twins, those (**A**) matching patient 1 or (**B**) matching patient 6 were re-simulated with an alternative dose composition and different dose amounts. Each row represents a different dose composition or dose amount that was simulated. Each column displays either the dose composition or the cellular kinetics of the TCR-engineered T cell subsets. Cellular kinetics of the digital twins treated with the original dose composition are replotted (gray curves) to provide a baseline for comparison to cellular kinetics resulting from alternative dosing simulations (colored curves, blue - T_scm_, green - T_cm_, purple - T_em_, or red - T_eff_). **C** Bar plots show persistence (% TCT in blood at day 365) as a function of percent cell composition for each of the four cell phenotypes. The bar plot height represents the average persistence metric across the 100 digital twins).

In addition to dose amount and patient-specific biological variability, dose composition may impact expansion and persistence of T cells^19–22^. To illustrate the impact of alternative dose compositions, we simulated a different dose composition in representative patients from the low (patient 1) and mid dose (patient 6) groups (Fig. 6). We used a T_scm_-enriched composition (85% T_scm_, 5% T_cm_, 5% T_em_, and 5% T_eff_) based on the hypothesis that T_scm_ enrichment drives better expansion and persistence. The cellular kinetics after treatment with the original dose composition are compared with those predicted after treatment with a T_scm_-enriched dose at two different dose levels. Furthermore, we performed a systematic variation of dose composition across all digital twins to relate dose composition to persistence (Fig. 6C).

The predicted cellular kinetics support the hypothesis that treatment with T_scm_ cells leads to greater overall expansion and persistence of the TCR-engineered T cells. For patient 6, infusion of a 10-fold lower dose (10^9^ cells) of T_scm_-enriched product is predicted to yield comparable overall cell expansion and persistence as infusion of 10^10^ cells of the original dosing material, which is mainly composed of T_em_ and T_eff_ cells (Fig. 6B). For patient 1, simulated infusion of the T_scm_-enriched material at the original dose level of 10^9^ cells increases T cell counts in blood by approximately 100-fold compared to the original dosing material, which is mainly composed of T_em_ and T_eff_ (Fig. 6A). The finding that greater T_scm_ composition in the infusion product leads to greater T cell abundance in these patients is consistent with our findings on T_scm_ parameters as critical determinants of cellular kinetics and persistence and is further supported by systematic variation of dose composition. We simulated 5000 unique random dose compositions and calculated the percent of TCT in the blood 365 days after infusion for each digital twin. Bar plots in Fig. 6C display this persistence metric averaged across all digital twins, binned by 5% intervals (Fig. 6C). When a dose composition contains 75% T_scm_, 80% of blood T cells are TCT at day 365. Similarly, if 75% of the dose composition is T_cm_, the persistence decreases and only ~50% of the blood T cells are TCT at day 365. Further, if 75% of the dose composition is either T_em_ or T_eff_, the persistence metric will further drop to ~38%. These findings could not be derived from a correlation analysis between dose composition and persistence of the TCR-engineered T cells from the observed clinical data in the E7 clinical trial (Supplementary Fig. 5). More generally, the results illustrate how the QSP model and digital twins can be used to explore optimization of dose composition and amount to achieve greater T cell persistence.

### Digital twins predict KRAS G12D T cell profiles in pancreatic cancer patients

**Fig. 7:**
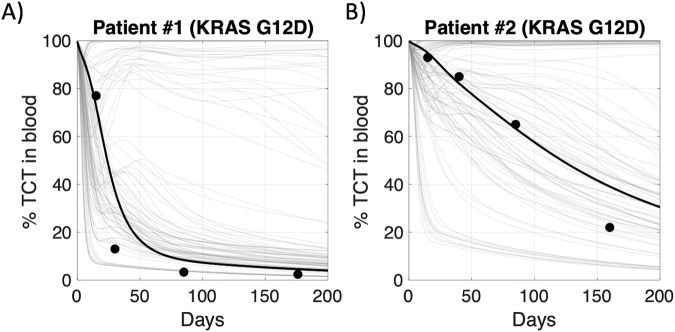
Predictive simulations of digital twins capture observed profiles in pancreatic cancer patients treated with TCR-engineered T cells targeting KRAS G12D. TCR-engineered T cells, as a percentage of total T cells in blood, for (**A**) patient #1 and (**B**) patient #2 are displayed across time. All 100 digital twins (gray curves) were re-simulated with the dose amount and dose composition outlined in the KRAS G12D clinical trial and plotted within the same graph. Black curves show the single digital twin that best recapitulate the observed data of each patient according to RMSE.

To explore the relevance of the QSP model structure and digital twins for other TCR-engineered T cell therapies and indications, we simulated the 100 digital twin virtual population at the same dose and composition used in a clinical study of KRAS G12D targeting TCR-engineered T cells in patients with metastatic pancreatic cancer (Fig. 7, see Supplementary Methods for details). The KRAS G12D clinical dataset was selected for this exercise as it is a publicly available dataset that provides a description of dose composition and dose amount, as well as the cellular kinetics of this therapy across time for both patients^17^. In the KRAS G12D clinical trial, both patients received a middle dose of ~10^10^ cells containing a majority of T_em_ cells, but patient #2 received a greater number of early memory cells. The different simulation curves reflect both the inter- and intra-patient variability represented in the digital twins, and interestingly, the clinical patient data generally corresponds to the highest density of simulated profiles. We further highlight the digital twin simulation that best matches the clinical patient’s data to illustrate the correspondence. These results support our broader findings on our model structure and the importance of T_scm_ and T_cm_ cells for persistence and suggest that the model and digital twins might help anticipate cellular kinetics and variability for TCR-engineered T cells targeting other antigens and indications.

## Discussion

The expansion and persistence of adoptively transferred T cell therapeutics have been linked to anti-cancer efficacy. However, understanding, predicting, and optimizing cellular kinetics remain a challenge, as numerous factors can impact cellular kinetics. In early clinical trials, dose levels range several orders of magnitude and manufacturing considerations make it difficult to supply patients with identical dose compositions. Additionally, patient heterogeneity can significantly impact cellular kinetics, via both the quality of the harvested and engineered T cells in autologous therapies and the interaction of the administered T cells and the host. Clinical trial data highlight characteristic behaviors such as expansion, contraction and persistence, but the most critical determinants of expansion and persistence are not well understood and determining which patients may achieve those cellular kinetics poses a significant challenge. Mechanistic modeling approaches offer a way to leverage biological knowledge to quantitatively deconvolute the mechanisms underlying data and provide insights and predictions about the biological processes behind diverse outcomes.

To better understand biological drivers of variability in cellular kinetic profiles, we developed a QSP model that describes T cell memory phenotypes across multiple physiologically relevant compartments, including blood, tumor, tumor-draining lymph nodes, and other healthy tissue. We include each of the aforementioned compartments in order to capture the distinct phases of cellular kinetics and represent the biological mechanisms that contribute to the observed dynamics. In particular, the tumor and TDLN represent sites of antigen-driven differentiation and proliferation, and the healthy tissue compartment is key to capturing margination and endogenous T cell expansion following lymphodepletion. A “reference virtual patient” parameterization of the generalized model captures the clinical trial data of four different TCR-engineered T cell phenotypes in the blood across three different dose amounts and various dose compositions. For each clinical patient, we further generated multiple digital twins that reproduce the patient’s observed T cell kinetics in response to treatment with their unique dosing material. Analysis and simulation of all digital twins allowed us to identify the biology most strongly influencing cellular kinetics and interpatient variability and to explore alternative dosing scenarios.

To our knowledge, this is the first systems model of T cell therapy that addresses TCR-engineered T cell therapy solid tumors, represents multiple memory subsets of T cells, and includes multiple tissue compartments. The latter is especially important for TCR-engineered T cells, for which trafficking to tumor-draining lymph nodes and tumor is required for antigen-induced cell expansion, differentiation, and tumor cell killing respectively. Furthermore, we believe this is the first use of patient-matched digital twins in the T cell therapy context.

Digital twins broadly refer to a model and accompanying parameterizations that capture the behavior of a “system” of interest for a single patient. Digital twins have been used across the fields of immunology and immunotherapy^23,24^, including to assess infectious disease outcomes^25^, to simulate the dose-response of bispecific antibodies in virtual clinical trials^16^, and to provide the control arm of clinical trials via disease progression modeling^26^. As the calibration data in this study came from a phase 1 clinical trial, the data included a limited number of subjects across multiple dose groups with high levels of variability. The digital twin approach has previously been shown to be useful to address these challenges^16^. Further, these digital twins provide a novel approach to investigating cellular kinetics profiles and predicting the impact of varying dose and phenotype ratios of TCR-engineered T cell therapies, through virtual simulation of alternate treatment protocols.

In this work, we used digital twins to provide quantitative, analysis-based hypotheses for why some patients show robust T cell expansion and long-term persistence while others do not. Analysis of digital twins stratified according to persister or non-persister outcomes suggests that the proliferation and trafficking of T_scm_ cells is an important determinant of persistence that differs from patient to patient. These parameters are interrelated as antigen-driven proliferation of cells occurs in tumor-draining lymph nodes, which requires trafficking from the blood. Additionally, proliferation of endogenous T cells was identified as impacting persistence of the TCR-engineered T cells, indicating competition between cell types. Finally, apoptosis of T_em_ cells impacts cellular kinetics by determining the persistence of the T_em_ subset. These results were consistent with analyses comparing parameter differences between dose groups and between individual patients.

Taken together, these analyses emphasize the potential of optimizing treatment regimens to maximize cell expansion and persistence. The predicted importance of endogenous T cell proliferation on engineered T cell persistence underscores the role of competition between the administered and endogenous T cells. Further, it highlights the value of lymphodepletion regimens, which are often administered prior to the TCR-engineered T cell therapy to lower T_endo_ cell counts prior to treatment^27–31^. Similarly, as T_scm_ proliferation was identified as an important determinant of cell kinetics and persistence, optimizing cytokine co-administration may improve cell expansion. In this study, IL-2 was administered and while there was no clear relationship between cell expansion and the amount of IL-2 dosed (data not shown), we implicitly captured the effects of IL-2 administration in the patient-specific proliferation rates. However, the importance of these proliferation rates in our analysis suggests other cytokines could be co-administered with T cell therapy including IL-7, IL-15, or IL-21 to facilitate the production of T_scm_ and T_cm_ cells, as these cells may offer greater persistence and therefore more robust anti-tumor activities in vivo across time^22,32–40^.

Our work additionally supports the hypothesis that altering the dose composition of T cell therapy can have a profound impact on the cellular kinetics across time. Simulated treatment of digital twins with a dose composed of primarily T_scm_ cells rather than T_em_ cells improved cell expansion and persistence. Again, this is consistent with the in silico exploration of mechanistic drivers of cellular kinetics that highlighted T_scm_ related parameters. Importantly, a simple correlation analysis between cell composition and persistence in the clinical data does not readily suggest a relationship between T_scm_ and persistence which could be due to different confounding sources of variability (Supplementary Fig. 5). Given the observed, complex relationship between dose amount, safety, and efficacy of T cell therapy in hematological malignancies^41^, altering the dose composition may provide a method of mitigating adverse events while still providing the necessary dose for efficacy.

While early work in CAR T cell therapies focused on establishing relationships between overall quantities of administered T cells and clinical response, recent work has acknowledged the key role of different phenotypes within the T cell products. Increasingly, preclinical and clinical studies have drawn correlations between stem-like capabilities of T cells and outcomes such as expansion, persistence, and efficacy^42–45^. Consistent with our model predictions and analysis for TCR-engineered T cell therapies, T_scm_ CAR T cells were recently shown to exhibit a proliferative advantage over conventional CAR T cells in in vitro and in vivo settings^9,22,32^. While research continues on optimizing ex vivo T cell processes to increase T_scm_ abundance, the model enables exploration of the predicted kinetics for dose compositions achieved, and can be used to help set minimum dose material requirements for desired kinetics.

Using digital twins to make prospective predictions assumes that the kinetics observed in the clinical patients in the HPV-16 E7 clinical trial are representative of T cell therapy more broadly, and that the model and key features of the digital twins are relevant to other T cell therapies and indications. We showed that a retrospective, predictive simulation of the digital twins captured the cellular kinetics observed for KRAS G12D TCR-engineered T cell therapy in pancreatic cancer patients. This work provides increased confidence in the utility of the QSP model and digital twins for simulation and exploration of other adoptive T cell therapies, even in other cancer indications. Further, it illustrates the applicability of the digital twin approach for future investigations. However, observed quantitative responses to T cell therapies are expected to be patient, therapy (e.g., target-antigen, affinity), and indication dependent.

In this work we focused on capturing autologous CD8 + T cells as the majority of TCR-engineered T cell therapies in clinical studies utilize CD8 + T cells. With appropriate data, the model can be modified to separately capture CD4+ and CD8 + T cell kinetics, as potentially both cell types influence therapeutic efficacy. Further, with appropriate data, the model could capture allogenic T cell therapies, which may exhibit some advantages to autologous T cells^46^. A limitation of this work is that we did not focus on tumor response, but rather on T cell expansion and persistence, which have been related to efficacy of CAR T therapies. Although the model includes equations and parameters for T-cell mediated tumor killing, absolute tumor size was not reported in the study and tumor-killing relies on the relative number of T cells to target cells^47,48^. Quantitative data on T cell exhaustion markers or gene expression, target antigen availability, and ideally engineered T cells in post-treatment tumor biopsy would also be valuable for linking cellular kinetics to tumor killing (i.e. therapeutic efficacy) for solid-tumor indications. Currently, we have modeled a theoretical “effective antigen load” that decays exponentially across time to represent various biological processes that might reduce the immune response as time progresses following treatment (Supplementary Fig. 6). This includes known mechanisms beyond tumor burden, such as T-cell exhaustion^49,50^, poor antigen presentation or immune escape mechanisms^51^ that have not been explicitly included in the model but are implicitly captured by our empirical representation. With appropriate datasets, future work could link cell kinetics, antigen availability, and tumor burden and response.

Despite limitations, the QSP model and digital twin methodology may have considerable applications beyond those presented herein. Currently, the QSP model integrates clinical data with mechanistic understanding in a unified and quantitative representation of cellular kinetics and variability for TCR-engineered T cell treatment in solid tumor indications. However, modification to address CAR T cell therapies or hematological malignancies is straightforward. Additionally, while beyond the scope of the current work, the digital twin methodology provides an opportunity for personalizing the model to patient characteristics and measurements (e.g. age, previous treatments, or baseline biomarker data) in order to predict and optimize treatment for each unique patient.

## Methods

### QSP model structure

We constructed a mechanistic QSP model composed of ordinary differential equations to describe the underlying cellular kinetic processes governing the in vivo dynamics of T cells during TCR-engineered T cell therapy in solid tumor indications. The model is designed to reproduce clinical measurements of the kinetics of different subsets of T cells, specifically: stem-like memory T cells (T_scm_), central memory T cells (T_cm_), effector memory T cells (T_em_), and effector T cells (T_eff_) from the TCR-engineered T cell therapy alongside endogenous T cells (T_endo_), following lymphodepletion and adoptive T cell transfer. As illustrated in Fig. 1, the model includes four physiologically relevant compartments – blood, tumor-draining lymph node (TDLN), a lumped compartment representing normal tissues including lymph nodes (Tissue/LN) and tumor. Lymphodepletion and adoptive T cell infusion are simulated by specifying baseline values of T_endo,_ T_scm,_ T_cm,_ T_em,_ and T_eff_ cells in the blood based on the extent of initial lymphodepletion and the dose amount of infused T cells.

Each T cell subset undergoes homeostatic proliferation and apoptosis, which can occur in blood, TDLN and Tissue/LN compartments. Due to the lack of clinical measurements in tumor and peripheral tissues and given the focus on circulating cell dynamics, for each cell subset, we use a common trafficking rate constant for all tissues, although the partition coefficient differs across tissues. Furthermore, while T_scm_ and T_cm_ cells can traffic into the tumor-draining lymph node, T_em_ and T_eff_ cells cannot, due to their lack of CD62L, which assists early-differentiated memory T cells extravasation into the lymph node environment^52–54^. Conversely, as effector cells, T_em_ and T_eff_ can traffic from blood to tumor. Within TDLN, all TCR-engineered T cells undergo antigen-driven proliferation and differentiation, with the exception of T_eff_ cells, as they are a terminally differentiated phenotype. Antigen load (antigen presented within the context of HLA) is represented as ‘tumor’ within the model and decreases following the start of the simulation to simulate the loss of antigen in TDLN after treatment (Supplementary Fig. 6). All compartment volumes and partition coefficients were based on literature values^55–65^. Further information on the model details, equations and parameter values are included in the Supplementary Methods.

### HPV-16 E7 TCR-engineered T cell therapy clinical data

For model calibration and digital twin generation, we used previously published data^15^. Briefly, in a first-in-human, phase 1 clinical trial, 12 patients were treated with T cells engineered with a TCR targeting HPV-16 E7 for the treatment of metastatic human papilloma virus-associated epithelial cancers. Patients were grouped into 3 separate cohorts, wherein patients received 10^9^, 10^10^, or 10^11^ engineered T cells in the low, medium and high dose cohorts, respectively. All patients were dosed a single time, at day 0, via intravenous infusion. Seven days prior to administration of the TCR-engineered T cell therapy, patients underwent a lymphocyte depletion regimen of cyclophosphamide (half of all patients received 60 mg/kg, the other six patients received a reduced dose of 30 mg/kg). All patients had received previous systemic anti-cancer treatments.

For the purposes of this study, two patients (patient IDs 2 and 11) were removed from the dataset due to lack of observed measurements. Reported measurements for the other 10 patients include cell concentrations (cell/mL) of T_scm_, T_cm_, T_em_, and T_eff_ TCR-engineered T cells, as well as T_endo_ cell concentration across time. The timepoints of measurement differ from patient to patient. When running patient-specific simulations, we simulated dosing with the reported dose amount as well as the reported dose composition of TCR-engineered T cell phenotypes for that individual patient.

### KRAS G12D TCR-engineered T cell therapy clinical data

To show the utility of the digital twin methodology for another TCR-engineered T cell therapy, we used clinical data described previously in a study of two pancreatic cancer patients who received KRAS G12D TCR-engineered T cell therapy^17^. Both patients received doses of CD8 + TCR-engineered T cells that were comparable in total count to the middle dose cohort in the HPV-16 E7 clinical trial. One patient received a dose of 14.8 × 10^9^ cells and the other patient received a dose of 29.6 × 10^9^ cells. Both doses contained a majority of effector memory T cells, and patient #2 received more central memory T cells than patient #1 (see Supplementary Table 2). For both patients, total TCR-engineered T cell kinetics data is reported as a percentage of the total number of T cells in the blood.

### Model calibration, digital twin generation

For model calibration and digital twin generation, we used the previously published data HPV-16 E7 TCR-engineered T cell therapy clinical data^15^. We selected this dataset for calibration as it is publicly available clinical data that includes dose amount and dose composition for each patient in addition to the cellular kinetics of each T cell phenotype across time. We first generated a “reference virtual patient” (Supplementary Fig. 1), a simulation that reasonably describes the cellular kinetics of each dose level in the blood following treatment with TCR-engineered T cells. Across three dose cohorts (low dose - 10^9^ cells, middle dose - 10^10^ cells, high dose - 10^11^ cells), simulation of the reference patient at each dose reproduces the cellular kinetics from patients in the corresponding dose cohort. See Supplementary Methods for more details. Using the reference virtual patient alone, it can be difficult to capture the individual patient profiles and explain the heterogeneity present within the HPV E7 clinical trial. Therefore, digital twins were generated (Supplementary Fig. 2) to match the TCR-engineered T cell memory phenotypes and endogenous T cell measurements for each individual patient in the trial. The digital twin methodology is a relatively new approach for calibrating QSP models when observed data is limited in a given dosing scenario and there is high degree of variability. It is preferable to other approaches (such as non-linear mixed effects modeling) in these situations; wherein a large number of mechanistic, biologically relevant parameters are calibrated to data containing a few observations at each time point, as often encountered in QSP models^16,24,66^. Detailed explanation of model calibration, digital twin analysis and a summary of all datasets is provided in Supplementary Methods.

### Reporting summary

Further information on research design is available in the Nature Research Reporting Summary linked to this article.

### Supplementary information

Supplementary Materials

Supplementary Table 1

Supplementary Table 2

Reporting summary

## Data Availability

The datasets generated and/or analyzed during the current study are available from the corresponding author on reasonable request.

## Code availability

The code utilized during the current study can be made available from the corresponding author on reasonable request.
