# Supplementary material for: Digital twins elucidate critical role of Tscm in clinical persistence of TCR-engineered cell therapy
Source: NPJ Syst Biol Appl. 2024 Jan 26;10:11. doi: 10.1038/s41540-024-00335-7 (PMC10817974; doi:10.1038/s41540-024-00335-7)
Supplement: Supplementary file 1 — Supplementary Materials [file 41540_2024_335_MOESM1_ESM.pdf]

Supplementary Material for "Digital twins elucidate critical role of T_{scm} in clinical persistence of TCR-engineered cell therapy"

Authors: Louis R. Joslyn[1#*], Weize Huang[1#], Dale Miles[1], Iraj Hosseini[1+], Saroja Ramanujan[1+]

[#]Co-first authors

[+]equal contribution authors

*Corresponding author: joslyn.louis@gene.com

Institutions:

[1] Genentech Inc., South San Francisco, CA.

Model Structure & Assumptions

Description of the QSP model

We develop a quantitative systems pharmacology (QSP) model to simulate the dynamic profiles of various T cell phenotypes during cell therapy treatments. The model is constructed to be consistent with underlying, theoretically relevant, biological mechanisms. The QSP model represents fundamental mechanisms of T cell biology across multiple physiologically relevant compartments for both endogenous and TCR-engineered T cells. These compartments include a central blood compartment, healthy tissues, tumor and tumor-draining lymph node compartments. The model includes biological mechanisms such as antigen-driven proliferation, antigen-driven differentiation, cell migration/trafficking, and homeostatic proliferation and apoptosis. The model is composed of ordinary differential equations that describe and predict the *in vivo* dynamics of T cell phenotypes including stem-like memory T cells (T_{scm}), central memory T cells (T_{cm}), effector memory T cells (T_{em}), and effector T cells (T_{eff}) from the TCR-engineered T cell therapy and endogenous T cells (T_{endo}) present in a patient during treatment. See sections below for greater details on biological mechanisms included in the model, as well as representation of tumor antigen, and lymphodepletion.

Biological mechanisms in QSP model

The following equations govern the general dynamics of TCR-engineered T cell subpopulations in the model. Briefly, these include homeostatic proliferation and apoptosis, trafficking/migration between compartments, antigen-driven proliferation and antigen-driven differentiation. The details and general equations for the T cells in each compartment are shown below.

Dynamics of TCR-engineered T cells in the blood

As an example, we display the general ordinary differential equation (ODE) for TCR-engineered T cells in the blood.

$$\begin{aligned}\frac{dT_{blood}}{dt} = Gen - Apop - TraffickToTiss(T_{blood} \leftrightarrow T_{tiss})\\ - TraffickToTumor(T_{blood} \rightarrow T_{tumor})\\ - TraffickToTDLN(T_{blood} \leftrightarrow T_{tiss})\end{aligned} \quad (1)$$

Where T_{blood} represents the number of TCR-engineered T cells (of a given T cell phenotype) in the blood with population kinetic terms for generation (Gen), apoptosis (Apop), and intercompartmental trafficking to other tissue types. Generation of T cells is modeled only to account for homeostatic expansion when the number of T cells is below homeostatic levels:

$Gen = k_{prolif} * T_{blood} * \left(\max\left(0, 1 - \frac{\frac{totTcells_{blood}}{V_{PB}}}{\frac{Tcellmax}{V_{PB}}}\right)\right)^2$, where k_{prolif} represents the proliferation rate for a given T cell phenotype (this value differs between T cell phenotypes, but does not change depending on the compartment this cell resides within) and $\frac{Tcellmax}{V_{PB}}$ represents

max capacity of T cells in the blood, as a concentration. Similarly, apoptosis of T cells is modeled to occur only when total T cell numbers exceed homeostatic levels. $Apop = k_{apop} * T_{blood} * \left(\max\left(0,1 - \frac{\frac{Tcellmin}{V_{PB}}}{\frac{totTcells_{blood}}{V_{PB}}}\right)\right)^2$, where k_{apop} represents the rate constant for apoptosis for each T cell phenotype and $\frac{Tcellmin}{V_{PB}}$ represents the minimum amount of T cells in the blood, as a concentration. The max and minimum values of T cells in tissues are the same values as $\frac{Tcellmax}{V_{PB}}$ and $\frac{Tcellmin}{V_{PB}}$, scaled by the partition coefficient for that tissue. In this way, TCR-engineered T cells and Tendo cells compete with one another as Tendo expand following lymphodepletion and TCR-engineered T cells expand following infusion and encounter with antigen. Additionally, effector T cells do not undergo homeostatic proliferation, as they are a terminal T cell population.

Trafficking of T cells

As shown in the kinetic terms above, trafficking can be unidirectional or bidirectional, depending on the compartment and T cell phenotype. As an example of trafficking dynamics, we display a general trafficking kinetic term for TCR-engineered T cells from the blood to the healthy tissue compartment. All cells can traffic in and out of the healthy tissue compartment.

$$TraffickToTiss(T_{blood} \leftrightarrow T_{tiss}) = k_{traffick} * V_{PB}\left(\frac{T_{blood}}{V_{PB}} * k_p - \frac{T_{tiss}}{V_{tiss}}\right) \quad (2)$$

The rate of trafficking for T cells to other tissues, takes a similar mathematical form, with the appropriate use of a tissue-specific partition coefficient (k_p). The partition coefficient is informed by baseline values of T cells in various tissues in healthy patients for each T cell phenotype (see parameter Table – Supplementary Table 1). This assumes that partitioning to tissue types will not differ due to TCR-engineering, however, the model has the flexibility to account for altered trafficking, should there be evidence for this behavior in cell therapies. The trafficking rate constant ($k_{traffick}$) differs across cell subpopulations (i.e. T_{scm} cells and T_{cm} cells will migrate at different rate constants), but does not differ for entry into the TDLN vs healthy tissues. However, we use a separate rate constant for tumor infiltration, as this rate should differ due to chemokines and inflammatory signals that may be present at the site of the tumor.
Once cells enter the tumor compartment, they do not migrate away from the tumor site. Only T_{em} and T_{eff} cells traffic to the tumor, whereas T_{em} and T_{eff} cells do not traffic into the tumor draining lymph node (i.e. only T_{scm} and T_{cm} cells can enter the lymph node), due to biological differences across these cell phenotypes.

Dynamics of TCR-engineered T cells in the tumor draining lymph node

As an example, we display the general ordinary differential equation (ODE) for TCR-engineered T cells in the tumor-draining lymph node (TDLN).

$$\frac{dT_{TDLN}^{x}}{dt} = Gen - Apop + Prolif - diff(T_{TDLN}^{x} \rightarrow T_{TDLN}^{x+1}) + diff(T_{TDLN}^{x-1} \rightarrow T_{TDLN}^{x}) + TraffickToTDLN(T_{blood}^{x} \leftrightarrow T_{Tissue}^{x}) \qquad (3)$$

Where T_{TDLN}^{x} is a general TCR-engineered T cell in the tumor draining lymph node. In the presence of antigen (as is the case in tumor draining lymph nodes), all but one of the TCR-engineered T cell phenotypes in the model (T_{scm}, T_{cm}, and T_{em}) undergo antigen-driven proliferation ($Prolif$) and differentiation ($diff$) within the tumor-draining lymph node. Antigen-driven proliferation is captured with the following michaelis-menten kinetics: $Prolif = k_{prolif_{atg}} * T_{TDLN}^{x} * \frac{tumor}{tumor+EC_{50_{prolif}}}$, where $tumor$ represents the tumor antigen that is present (see Tumor & Antigen Representation section below) and $EC_{50_{prolif}}$ represents the cell's responsiveness to that antigen in the form of a half-saturation term. Antigen-driven proliferation is similarly captured as a unidirectional reaction rate: $diff(T_{TDLN}^{x} \rightarrow T_{TDLN}^{x+1}) = k_{convX} * T_{TDLN}^{x} * \frac{tumor}{tumor+EC_{50_{conv}}}$, where k_{convX} represents the differentiation rate constant of TCR-engineered T cell *x* to TCR-engineered T cell *x+1* (e.g. T_{cm} cells to T_{em} cells) and $EC_{50_{conv}}$ represents the T cell (T_{TDLN}^{x}) likelihood of differentiation in the presence of antigen, formulated as a half-saturation term.

As a terminal T cell subpopulation, T_{eff} cells do not proliferate nor differentiate into another T cell phenotype. T_{endo} cells do not exhibit antigen-dependent proliferation or differentiation. As previously stated in the trafficking section above, only T_{scm} and T_{cm} cells have bidirectional trafficking in the TDLN, whereas T_{em} and T_{eff} cells do not migrate into the TDLN.

Tumor & antigen representation

We have modeled a theoretical ‘effective antigen load’ (referred to as $tumor$ in the above sections and following equations) to represent tumor antigen in the tumor draining lymph node and tumor across time. Antigen load drives antigen-driven proliferation and differentiation mechanisms in the model. Consistent with other models of antigen load [1–3], this antigen decreases relatively quickly across time (see Supplementary Figure 6) and is modeled to decay exponentially to represent various biologically processes that might reduce the immune response as time progresses following treatment. These include, but are not limited to, tumor burden size, T -cell exhaustion, poor antigen presentation, or immune escape. Instead of independently and mechanistically capturing these phenomena within our model, our empirical representation implicitly captures the combined effects of each process. $tumor$ growth and decay are captured with the following differential equation:

$$\frac{dtumor}{dt} = tumorGrowth - tumorKill \tag{4}$$

where

$$tumorGrowth = kprolif_{tumor} * tumor * \max\left(0, 1 - \frac{tumor}{cc}\right)^2 \tag{5}$$

$$tumorKill = kinhib_{tumor} * tumor * \frac{T_{em}^{tumor} + T_{eff}^{tumor}}{\left(T_{em}^{tumor} + T_{eff}^{tumor}\right) + kill_{50}} \tag{6}$$

Where $kprolif_{tumor}$ and $kinhib_{tumor}$ represent growth and decay rate constants for the tumor/antigen, accounting for nutrient availability and other immune pressures that have not been directly modeled at this time. Additionally, we include the model structure for the relative

number of T_{em} and T_{eff} at the site of the tumor to influence the killing of tumor cells, formulated as a Michaelis Menten term, but at this time, these cells have very little impact due to lack of clinical data to inform the mechanistic relationship between cell numbers and relative cell killing (we set $kill_{50}$ to a small value for cells/mL, see Supplementary Table 1).

With quantitative data on T cell gene expression or exhaustion markers in a tumor draining lymph node, the model could include more mechanism on the relationship between cellular kinetics and antigen load. Further still, counts of TCR-engineered T cells at the site of the tumor post-treatment, as well as tumor burden at treatment would be valuable for linking cellular kinetics to tumor killing and representing the tumor size more appropriately within the model.

Dosing & lymphodepletion

In the model, we simulate dosing by changing the initial conditions of each of TCR-engineered T cell phenotypes in blood according to the exact dose that was provided in each of the clinical trials (HPV-16 E7 clinical trial and KRAS G12D in pancreatic cancer patients). All digital twins are simulated with the same dose amount for the clinical patient whose cellular kinetics they best match. See sections below for details on the exact dose amount and dose composition for each clinical trial. We simulate lymphodepletion by lowering the count of endogenous T cells within the model at the start of the simulation to levels that are typically observed following lymphodepletion chemotherapy regimens (approximately 10 cells/uL for cells in the circulation and ~90% cell depletion for cells in other tissues [4]). Homeostatic proliferation mechanisms

within the model drive T_{endo} cell counts back up toward the original baseline as TCR-engineered T cells expand, leading to competition dynamics between these cell types.

Dose-dependent expansion

During initial model calibration, digital twin generation and analysis, we identified a relationship between dose amount and two terms in the model. First, the total capacity of T cells in the system appeared to depend on the dose provided in that high dose patients received a total number of TCR-engineered T cells that is greater than the typically assigned baseline values for T cell capacity in the blood (10^9 cells). We model the dose dependence with an initial assignment formulated as a V_{max} model to change the total capacity of T cells in the blood ($Tcellmax$) based on total dose (tot_{dose}):

$$Tcellmax = base_b * \left(1 + V_{dose} * \frac{tot_{dose}}{tot_{dose} + EC50_{dose}}\right) \quad (7)$$

Where $base_b$ is a constant set to 10^9 cells and V_{dose} is maximum contribution of dose to carrying capacity to represent the biological point at which cells can no longer proliferate, even in situations of even higher dosing. As $Tcellmax$ is scaled by a tissue-specific partition coefficient and then used in other tissues to drive homeostatic proliferation and apoptosis, we only need to do this single initial assignment to account for homeostatic mechanisms that might exhibit dose-dependence across the system.

Secondly, the expansion of T_{scm} cells appear to be dose dependent (see Supplementary Figure 1A) and the model was unable to reproduce expansion of this cell population across multiple dose groups (data/figures not shown) without including a dose dependent term. Therefore, we alter the antigen-driven proliferation term that is used for all other TCR-engineered T cell phenotypes (as shown in above sections) in the following manner:

$$Prolif_{scm} = k_{prolif_{atgscm}} * T_{TDLN}^{scm} * \frac{tumor}{tumor + EC50_{prolif}^{scm}} * \left(1 + V_{dose}^{scm} * \frac{tot_{dose}}{tot_{dose} + EC50_{dose}^{scm}}\right) \quad (8)$$

Where the dose dependence of antigen-driven T_{scm} cell proliferation is formulated similarly to the initial assignment for Tcellmax, with T_{scm} cell specific parameters: V_{dose}^{scm} and $EC50_{dose}^{scm}$. Once we implemented these dose-dependencies within the model framework, we were able to capture data across multiple dose group and multiple T cell phenotypes with a single reference virtual patient (Supplementary Figure 1B; see sections below for details.)

Clinical Data

HPV-16 E7 TCR-engineered T cell therapy clinical data

For model calibration and digital twin generation, we used previously published data [5]. Briefly, in a first-in-human, phase 1 clinical trial, 12 patients were treated with T cells engineered with a

TCR targeting HPV-16 E7 for the treatment of metastatic human papilloma virus-associated epithelial cancers. Patients were grouped into 3 separate cohorts, wherein patients received 10^9 , 10^{10}, or 10^{11} engineered T cells in the low, medium and high dose cohorts, respectively. All patients were dosed a single time, at day 0 via intravenous infusion. Seven days prior to administration of the TCR-engineered T cell therapy, patients underwent a lymphocyte depletion regimen of cyclophosphamide (half of all patients received 60 mg/kg, the other six patients received a reduced dose of 30 mg/kg). All patients had received previous systemic anti-cancer treatments.

For the purposes of this study, two patients (patient IDs 2 and 11) were removed from the dataset due to lack of observed measurements. Reported measurements for the other 10 patients include cell concentrations (cell/mL) of T_{scm}, T_{cm}, T_{em}, and T_{eff} TCR-engineered T cells, as well as T_{endo} cell concentration across time. The timepoints of measurement differ from patient to patient. When running patient-specific simulations, we simulated dosing with the reported dose amount as well as the reported dose composition of TCR-engineered T cell phenotypes for that individual patient.

KRAS G12D TCR-engineered T cell therapy clinical data

To validate the model and show the utility of the digital twin methodology for another TCR-engineered T cell therapy, we used clinical data described previously in a study of two pancreatic cancer patients who received KRAS G12D TCR-engineered T cell therapy [6]. Both patients received doses of CD8+ TCR-engineered T cells that were comparable in total count to the

middle dose cohort in the HPV-16 E7 clinical trial. One patient received a dose of 14.8 x 10^9 cells and the other patient received a dose of 29.6 x 10^9 cells. Both doses contained a majority of effector memory T cells, and patient #2 received more central memory T cells than patient #1 (see Supplementary Table 2). For both patients, total TCR-engineered T cell kinetics data is reported as a percentage of the total number of T cells in the blood.

Model calibration, digital twin generation, & analyses

QSP model calibration: identifying a "reference virtual patient"

Successful model calibration is defined as the identification of a single mathematical structure and associated parameter estimates that reasonably describe both the underlying biological processes and observed measurements of cells in the blood following TCR-engineered T cell therapies. Following model construction, we calibrated our QSP model simultaneously to each of the three dose cohorts in the HPV-16 E7 clinical trial. We calibrated our QSP model (Figure 1) using gQSPSim, a graphical user interface MATLAB application that performs key steps in the QSP model development process including model calibration using global optimization methods [7].

We defined a "reference virtual patient" that reasonably describes the cellular kinetics in the blood following treatment with TCR-engineered T cells. Specifically, we calibrated to median values for each TCR-engineered T cell phenotype and endogenous T cell phenotype across time. Due to variation in sampling timepoints across the patients, we selected median cell

concentration values at days 0, 75, 150, and 250 post-infusion for the low dose cohort, days 0, 8, 44, and 200 post-infusion for the middle dose cohort, and days 0, 8, 34, 100, and 250 for the high dose cohort. Once we identified the reference virtual patient, we then re-simulated this patient for each of the dose compositions that were provided to each patient across all the dose cohorts. Supplementary Figure 1 displays the reference virtual patient simulations overlayed on clinical data.

We developed a representative "reference virtual patient" that recapitulates the overall quantitative data on multiphasic cellular kinetics after therapy with T cells engineered to target HPV-16 E7 in epithelial cancer patients (Supplementary Figure 1B). Across three dose cohorts (low dose - 10^9 cells, middle dose - 10^{10} cells, high dose - 10^{11} cells), simulation of the reference patient at each dose reproduces the cellular kinetics from patients in the corresponding dose cohort. For the low dose group, T_{scm}, T_{cm}, T_{em}, and T_{eff} cell populations all traffic quickly out of the blood, a process generally referred to as margination, after which blood cell concentrations continue to wane over time. In the low dose group, neither the observed data nor the reference virtual patient suggests expansion of the therapy in the blood. Additionally, both the observed and simulated data show an endogenous T cell population that expands quickly following lymphocyte depletion and then rebounds to a steady state. For the middle dose group, clinical and simulated profiles show that while T_{cm} and T_{em} cell populations exhibit similar dynamics with initial margination and a subsequent decline over time, T_{scm} and T_{eff} cells exhibit cellular expansion following margination. Finally, at the high dose, both the clinical and simulated profiles exhibit similar dynamics to the middle dose group, but with an apparently greater

relative expansion of the T_{scm} cell population. Together, these results verify that simulation of the reference patient captures the dynamics of each TCR-engineered T cell subset measured, as well as the endogenous T cells, as a function of T cell dose (Supplementary Figure 1B).

Digital twin generation

Using the reference virtual patient alone, it can be difficult to capture the individual patient profiles and explain the heterogeneity present within the HPV E7 clinical trial. The small trial size and large heterogeneity of cellular responses between patients pose some unique challenges. The size of each dose cohort is very small, with only two patients in the low dose group, three patients in the middle dose group, and five patients in the high dose group. Additionally, there is notable interpatient variability in the cellular kinetics within and across dose cohorts. This heterogeneity is most pronounced in the middle dose cohort, but is observed across all dose cohorts (Supplementary Figure 1A).

The digital twin workflow is shown in Supplementary Figure 2. Digital twins were generated for the patients in the HPV-16 E7 clinical trial, using the TCR-engineered T cell memory phenotypes and endogenous T cell observations for each patient. In total, 10 digital twins were selected for each of the 10 patients, generating a digital twin population of 100 simulations that represent the cellular kinetics of the patients across the phase 1 clinical trial. The general workflow for generating and utilizing digital twins follows three main steps:

1. **Generate a set of digital twins for each clinical patient.**

The first step of the digital twin generation workflow contains two main sub-components. First, we generate many digital twin candidates, then filter through those candidate digital twins to find the simulations that best match each patient. Each individual sub-step is outlined below.

a. *Generate numerous candidate digital twins.* We simulated the QSP model with 10,000 parameter combinations within the original parameter space for calibration. We varied 13 parameters representing proliferation, differentiation and trafficking for each of the cell populations.

b. *For each patient, select the n=10 best simulations that match the data*. Looping through the total set of candidate digital twins, choose n=10 simulations according to an objective function evaluation. In this case, our objective function was specified as the sum of the squared logarithmic error for each observation, for each T cell phenotype. Our total set of 100 digital twins consists of 10 digital twins for each of the 10 patients. See Supplementary Table 1 for the baseline parameter values across the digital twins.

2. **Compare digital twins with an additional dataset**

The next step in a digital twin generation workflow is to compare predictions with these digital twins to an additional dataset. We compare the digital twins that were generated based on HPV-16 E7 clinical trial data against KRAS G12D clinical trial data. We compare the predictive digital twin simulations against TCR-engineered T cell kinetics data in Figure 7. The comparison of the digital twins provides increased confidence for

further predictive simulations using these parameter sets in future virtual clinical trials or other analysis.

3. **Perform various analysis with digital twins**

Unlike other stratification-based or virtual cohort approaches, once digital twins have been generated, subsequent analysis at an individual level can be performed. In our work, we utilize the digital twin simulations to predict the impact of patient-specific variability on cellular kinetics, and isolate differences in parameter space from patient to patient. Like virtual cohort approaches, we additionally analyze differences between the different dose cohorts, using our set of digital twins that match patients within a single cohort to act as a 'virtual cohort' to be compared against the digital twins of another dose cohort. Finally, we predict the impact of changing the dose composition on the cellular kinetics by re-simulating patients with different dose amounts and dose compositions.

Model environment and analysis

Model code, digital twin generation, calibration, preliminary data analysis, and figure generation is written in MATLAB (R2020b). Model structure was created in SimBiology. ODEs are solved using MATLAB's ode45 solver. Initial model calibration was performed using the gQSPSim application [7]. Parameter space analysis, including density plot generation, principal

component analysis, and global sensitivity analysis was implemented in R (v 4.1.0), using tidyverse, ggridges, epiR and ggpubr packages.

PCA analysis to identify drivers of variability across dose groups

To identify drivers of the variable cellular kinetics across dose groups, we performed principal component analysis (PCA) on the parameter space of the digital twin simulations. As PCA identifies combinations of underlying parameters that best explain the variability across the digital twins, this method provides an opportunity to explore mechanistic differences driving differing kinetics in the different dose cohorts.

As Figure 4 shows, there is a slight delineation across dose groups in the parameter space of the digital twins, with minimal overlap along the first principal component (PC1) between the low and high dose groups (tan vs. orange dots, Figure 4) and the mid-dose group overlapping both in the intermediate range. To examine the biology driving these differences, we examined the parameters with the strongest contributions to the first two principal components, PC1 and PC2 (Supplementary Figure 3). Parameters that govern proliferation and trafficking of T cells with an early memory phenotype (T_{scm} or T_{cm} cells) were important contributors to PC1, and thus to the differential kinetics between dose cohorts. Parameters driving PC2, specifically effector T cell (T_{eff}) apoptosis rates and parameters governing endogenous T cell (T_{endo}) behavior further contributed to differences between the cohorts. Note, dose amount and dose compositions were not provided as a feature for the PCA although consistent differences between cohorts in key

biological parameter values could reflect indirect effects of dose (e.g., dose-dependence of proliferation rate). Finally, the lack of dense clustering within each dose group in PCA space (as shown in Figure 4) suggests potential differences among patients, even within the same dose group.

Global Sensitivity Analysis

Mathematical modeling approaches that employ a single parameterization or utilize local sensitivity analyses typically suffer from an inability to accurately capture the full range of variability that might be present in experimental datasets. By sampling across a multi-dimensional parameter space, modelers can find multiple parameterizations that can explain the variability within the data (i.e., digital twin generation – as described above – is one such approach). Further, by varying parameter values across reasonable ranges, one can employ statistical techniques to assess the sensitivity of model outcomes to various parameterizations and therefore predict the relative importance of different biological mechanisms for both simulated and observed outcomes. These statistical techniques are generally referred to as sensitivity analyses [8,9].

We quantify the importance of each biological mechanism involved in cellular kinetics (and more specifically, persistence in our persister vs non-persister analysis) by finding correlations between model parameters and outputs. Correlations between specific model outputs (such as persistence, total T cell counts, or other model outputs) and parameters were determined by using Partial Rank Correlation Coefficient (PRCC), where a value of -1 denotes a perfect negative correlation between model output and a parameter and a value of +1 denotes a perfect positive

correlation between the two. For more information on these techniques, Marino et al. completed a review of various statistical tests available to assess significance of PRCC [8] and Renardy et al. performed a review of sampling schemes that can be paired with global sensitivity analyses [9].

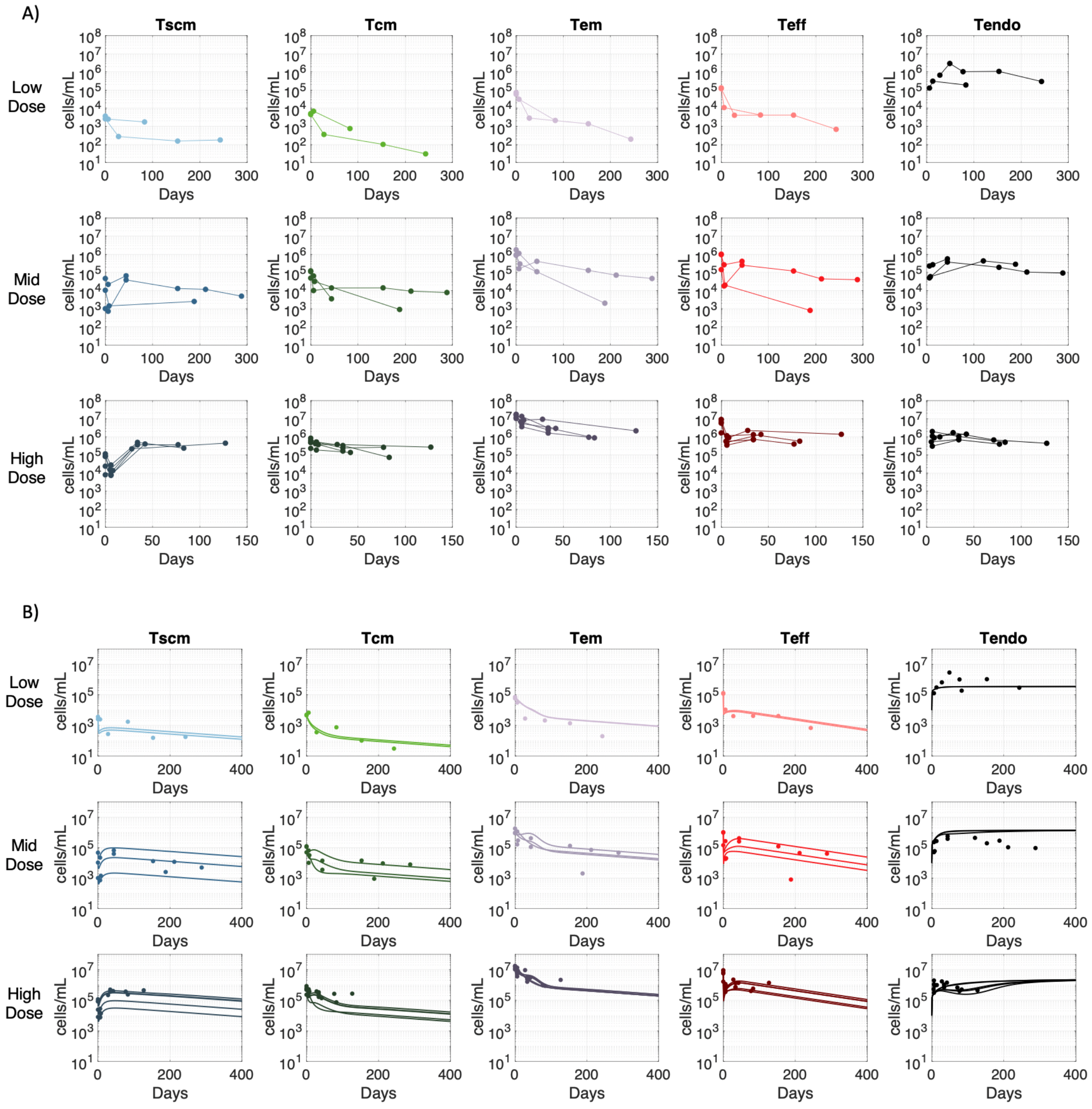

Supplementary Figure 1: Reference virtual patient captures clinical data on circulating T cell concentrations from the E7 T cell therapy clinical trial. *(A) Clinical observations, with each trajectory and circle markers representing one patient from the clinical trial across 3 dose groups (separate rows – 10^9, 10^{10}, 10^{11} infused T cells per group). (B) Reference virtual patient captures the observed data for T_{scm}, T_{cm}, Tem, T_{eff}, and T_{endo} cells across 3 dose groups (separate*

rows – 10^9, 10^{10}, 10^{11} infused T cells per group). Each separate line represents the reference virtual patient simulated with the dose composition for each clinical patient in that dose group.

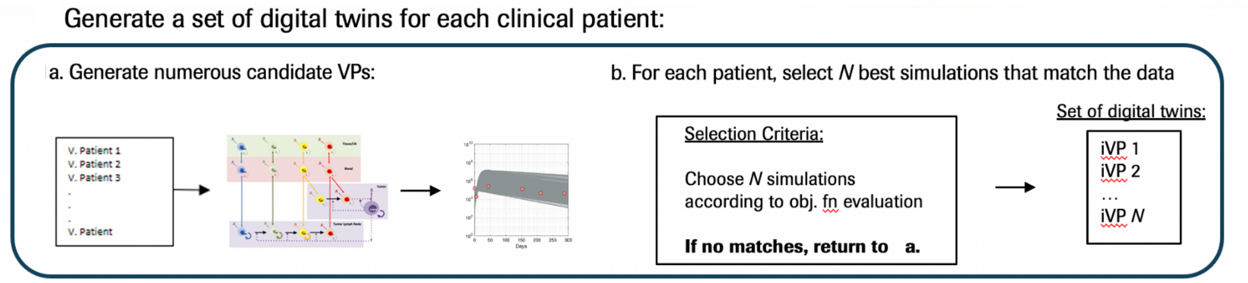

Supplementary Figure 2: Sample workflow for digital twin generation prior to downstream analysis. *A) Generate numerous candidate virtual patients via simulations of unique parameter combinations across parameter space. B) In an iterative manner, select the best simulations that match individual patient data thereby creating a set of digital twins for each patient.*

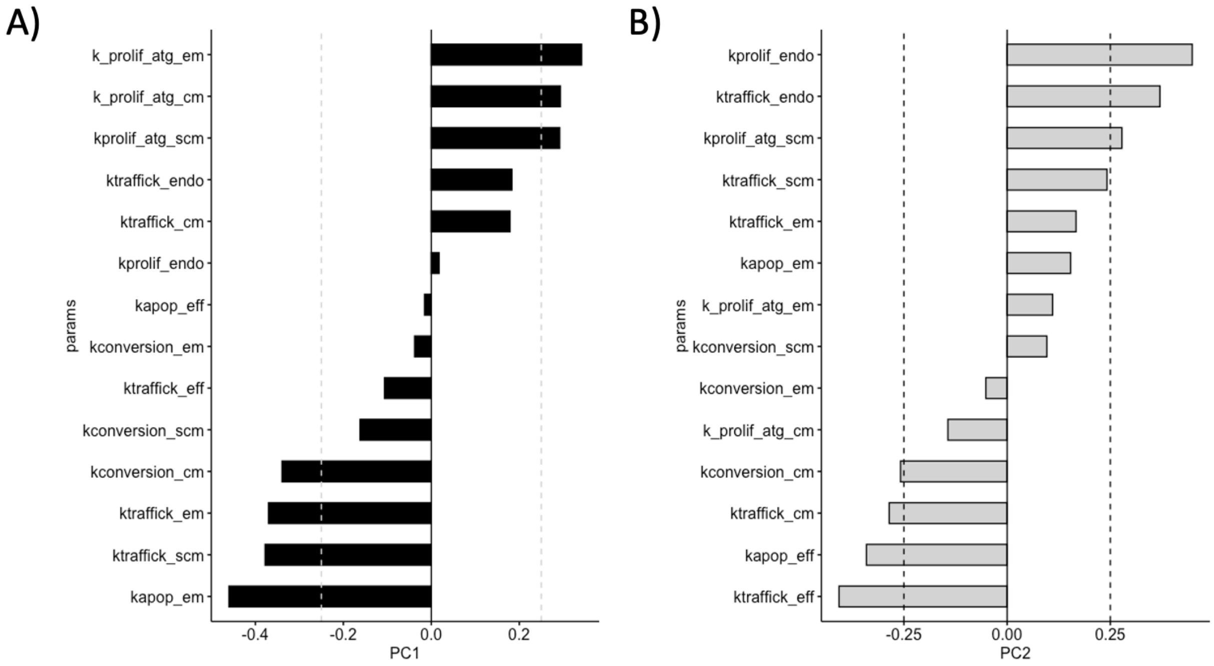

Supplementary Fig 3: ***Loading values for each model parameter (i.e. the contribution of each parameter) corresponding to either PC1 (A) or PC2 (B).***

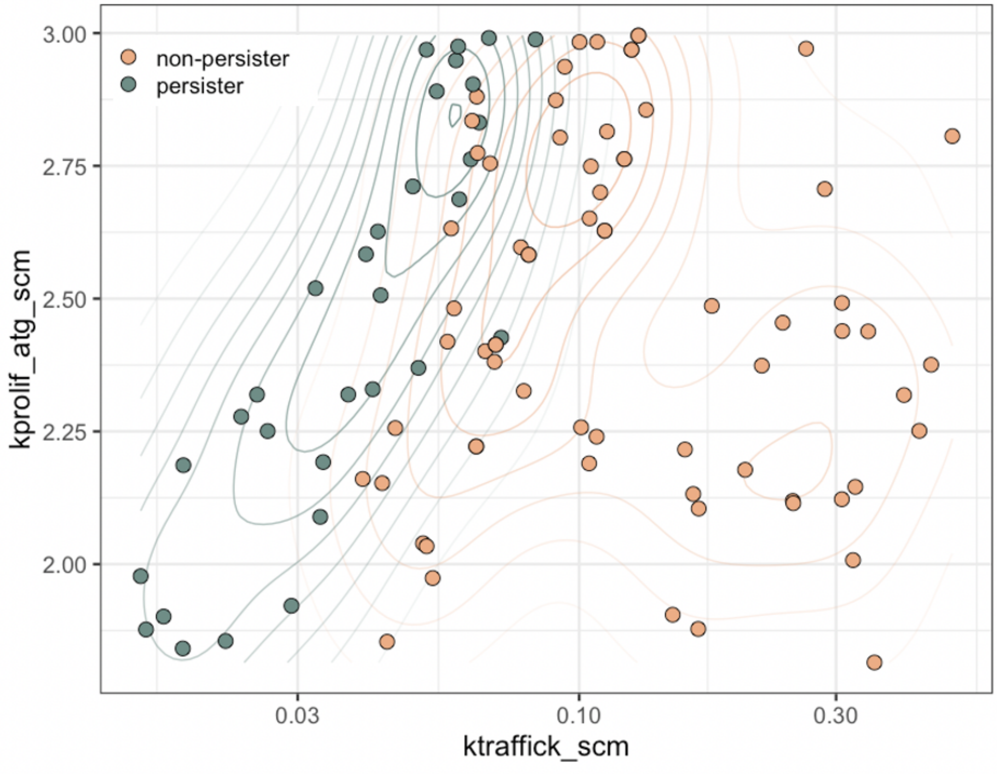

Supplementary Fig 4: Bivariate plot for comparison of parameter space for persisters vs non-persisters. *Along the x axis, the sampled parameter space for trafficking of Tscm cells is displayed and the parameter space for antigen-driven proliferation of Tscm cells is displayed along the y axis. Teal represent parameter combinations whose digital twins were persisters and peach represents non-persisters.*

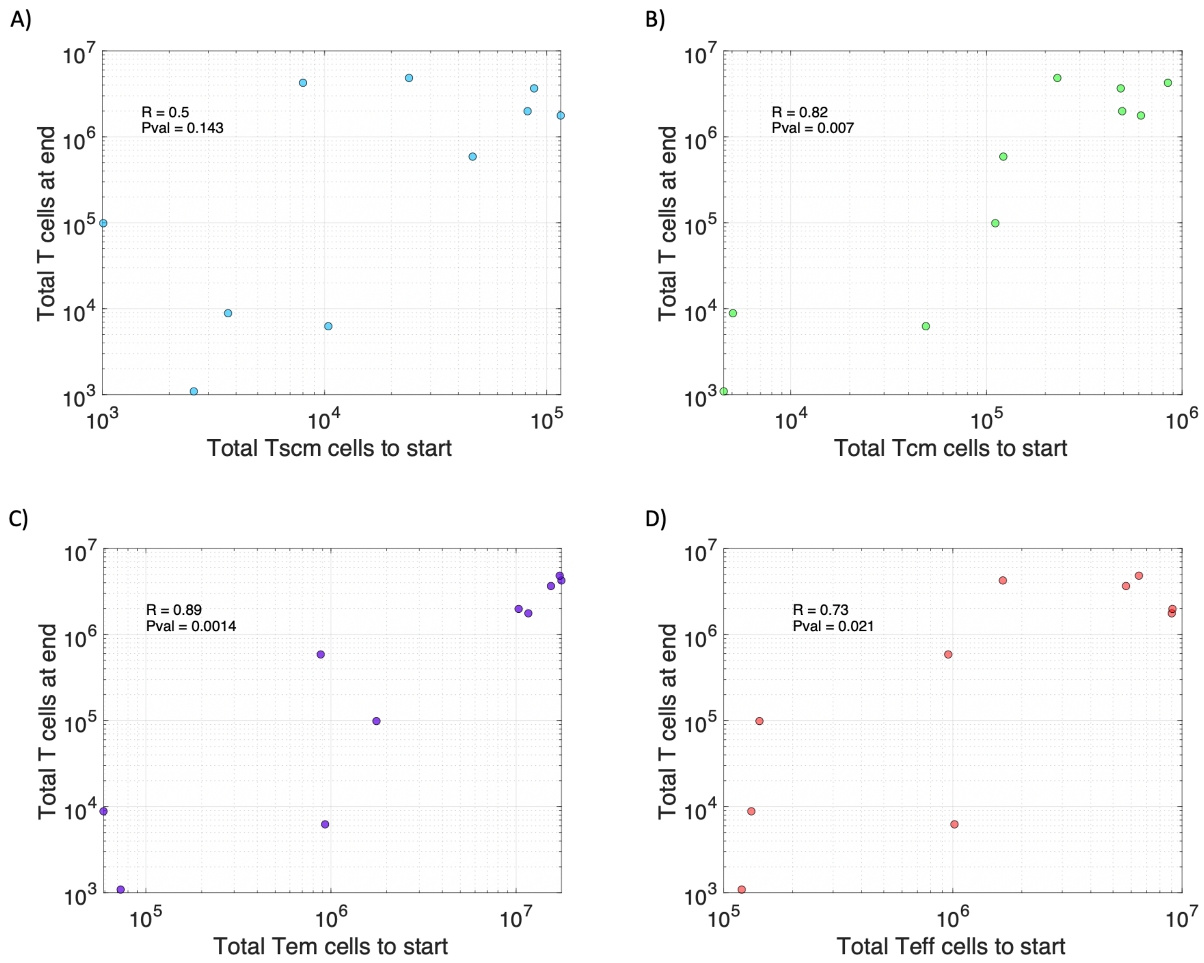

Supplementary Figure 5: *Relationship (Pearson correlation) between T cell phenotype (A – T_{scm} cells, B – T_{cm} cells, C – T_{em} cells, and D – Teff cells) at dose (logscale) and total T cells (logscale) at final time point in HPV16-E7 clinical trial.*

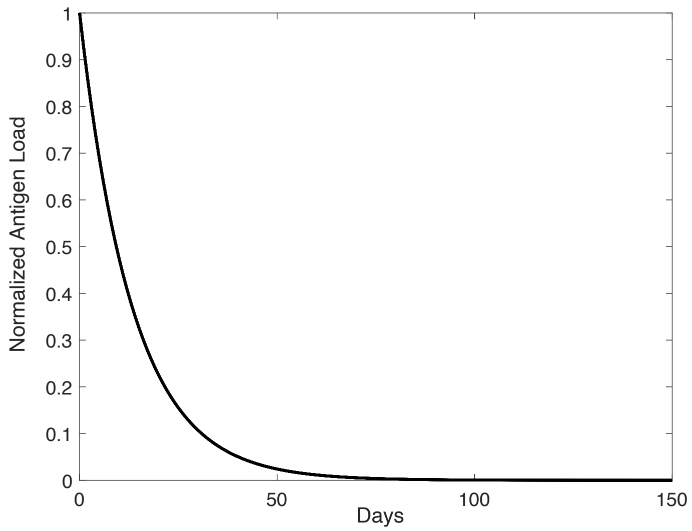

Supplementary Figure 6: *Reference virtual patient antigen load across time.*

Supplementary References

1. Joslyn LR, Linderman JJ, Kirschner DE. A virtual host model of Mycobacterium tuberculosis infection identifies early immune events as predictive of infection outcomes. J Theor Biol. 2022; 111042. doi:https://doi.org/10.1016/j.jtbi.2022.111042
2. Joslyn LR, Pienaar E, DiFazio RM, Suliman S, Kagina BM, Flynn JAL, et al. Integrating non-human primate, human, and mathematical studies to determine the influence of BCG timing on H56 vaccine outcomes. Front Microbiol. 2018;9. doi:10.3389/fmicb.2018.01734
3. Marino S, Kirschner D. A Multi-Compartment Hybrid Computational Model Predicts Key Roles for Dendritic Cells in Tuberculosis Infection. Computation. 2016;4: 39. doi:10.3390/computation4040039
4. Singh A, Parmar K, Mugundu G. CHARACTERIZATION OF CAR-T CELLULAR KINETICS AND EFFICACY IN PATIENTS WITH SOLID TUMORS IN PRESENCE OR ABSENCE OF PRIOR LYMPHODEPLETION CHEMOTHERAPY USING A PBPK-PD MODEL. CLINICAL PHARMACOLOGY & THERAPEUTICS. WILEY 111 RIVER ST, HOBOKEN 07030-5774, NJ USA; 2022. pp. S66–S66.
5. Nagarsheth NB, Norberg SM, Sinkoe AL, Adhikary S, Meyer TJ, Lack JB, et al. TCR-engineered T cells targeting E7 for patients with metastatic HPV-associated epithelial cancers. Nat Med. 2021;27: 419–425. doi:10.1038/s41591-020-01225-1
6. Leidner R, Sanjuan Silva N, Huang H, Sprott D, Zheng C, Shih Y-P, et al. Neoantigen T-Cell Receptor Gene Therapy in Pancreatic Cancer. New England Journal of Medicine. 2022;386: 2112–2119. doi:10.1056/NEJMoa2119662
7. Hosseini I, Feigelman J, Gajjala A, Susilo M, Ramakrishnan V, Ramanujan S, et al. gQSPSim: A SimBiology-Based GUI for Standardized QSP Model Development and Application. CPT Pharmacometrics Syst Pharmacol. 2020/03/10. 2020;9: 165–176. doi:10.1002/psp4.12494
8. Marino S, Hogue IB, Ray CJ, Kirschner DE. A methodology for performing global uncertainty and sensitivity analysis in systems biology. Journal of Theoretical Biology. 2008. pp. 178–196. doi:10.1016/j.jtbi.2008.04.011
9. Renardy M, Joslyn LR, Millar JA, Kirschner DE. To Sobol or not to Sobol? The effects of sampling schemes in systems biology applications. Math Biosci. 2021;337: 108593. doi:https://doi.org/10.1016/j.mbs.2021.108593
